# Ameliorative Role of Ethanolic *Nigella sativa* Seed Extract in Chlorpyrifos‐Induced Neurotoxicity and Testicular Toxicity

**DOI:** 10.1155/vmi/9865544

**Published:** 2026-09-25

**Authors:** Dhanya R. Rai, Reena Sherin Parveen, Veena Nayak, Sandhya Kumari, Vinutha R. Bhat, Harshini, Mohandas Rao K. G.

**Affiliations:** ^1^ Department of Pharmacology, Kasturba Medical College, Manipal Academy of Higher Education, Manipal, India, manipal.edu; ^2^ Department of Reproductive Sciences, Kasturba Medical College, Manipal Academy of Higher Education, Manipal, India, manipal.edu; ^3^ Department of Biochemistry, Kasturba Medical College, Manipal Academy of Higher Education, Manipal, India, manipal.edu; ^4^ Department of Basic Medical Sciences, Kasturba Medical College, Manipal Academy of Higher Education, Manipal, India, manipal.edu

**Keywords:** atropine, Morris water maze, neuroprotective, *Nigella sativa*, organophosphate insecticide

## Abstract

**Background and Aim:**

Chlorpyrifos, an organophosphate (OP) pesticide, is used worldwide in agriculture as an insecticide; its exposure has been linked to neurological disorder, endocrine disruption, cardiovascular disease, etc. *Nigella sativa* (NS), also known as black seed or black cumin, has been a part of traditional medicine and is used to treat a wide range of ailments from digestive and respiratory problems to topical skin disease. This study aims to investigate the potential protective role of the ethanolic seed extract of NS on chlorpyrifos‐induced neurotoxicity and testicular toxicity.

**Materials and Methods:**

A total of 36 male Wistar rats were divided into six groups, which included Group 1 as Control group, Group 2 as CPF 7.5 mg/kg, Group 3 as CPF 7.5 mg/kg + ATR 10 mg/kg, Group 4 as CPF 7.5 mg/kg + NS 2.5 mg/kg, Group 5 as CPF 7.5 mg/kg + NS 5 mg/kg, and Group 6 as CPF 7.5 mg/kg + NS 10 mg/kg. NS and CPF in their respective doses were administered orally, and ATR was given as intraperitoneal injection every day for 28 days. Neurotoxicity was assessed by measuring oxidative stress markers (malondialdehyde [MDA], a marker of lipid peroxidation), antioxidant status (reduced glutathione [GSH]), inflammatory markers (interleukin‐1β [IL‐1β]), and acetylcholinesterase (AChE) activity. Cognitive function was assessed using the Morris Water Maze test. Testicular toxicity was evaluated through sperm count and histopathology assessment.

**Results:**

The treatment with NS at a dose of 10 mg/kg showed neuroprotective effect through elevation in AChE activity, significant decrease in IL‐β levels, dose‐dependent decrease, and increase in MDA and GSH levels, respectively, and improved spatial memory as evidenced by increase in escape latency and the number of entries in the Morris water maze test. However, there was no significant effect in testicular toxicity.

**Conclusion:**

The results of the present study indicate that the ethanolic seed extract of NS has significant neuroprotective activity against CPF‐induced neurotoxicity, mainly at the dose of 10 mg/kg, as portrayed by the improvement in AChE activity and cognitive performance combined with its anti‐inflammatory effect through a decrease in IL‐1β levels and antioxidant effect through increase in GSH and reduction in MDA levels. However, the NS extract did not show any significant protective effect against CPF‐induced testicular toxicity. These results indicate the potential therapeutic role of NS in mitigating OP‐induced neurological damage, justifying further studies to elucidate its mechanisms and extended benefits.

## 1. Introduction

Pesticides are extensively used worldwide to eradicate weeds, fungi, rodents, and insects, and among them, the most prevalent organophosphate (OP) pesticide used worldwide in agriculture is chlorpyrifos (CPF) [1]. Neurological disorders, endocrine disturbance, cardiovascular disease, haematological malignancies, anomalies in development and behaviour, genotoxicity, histopathological aberrations, immunotoxicity, and oxidative stress have all been related to the toxicity of CPF. This is caused by the irreversible suppression of the enzyme acetylcholinesterase (AChE) that hydrolyses the neurotransmitter acetylcholine (ACh), leading to an excessive build‐up of ACh at both nicotinic and muscarinic cholinergic receptors, leading to toxicity [2–5]. In addition to lipid peroxidation (LPO), reactive oxygen species (ROS) generated by oxidative stress of OPs cause damage to cellular proteins and DNA, leading to tissue toxicity [6–9]. This led to the growing concern that human exposure to CPF will lead to harmful health consequences.

Atropine (ATR), an anticholinergic alkaloid, is the mainstay of treatment for OP poisoning. By acting at muscarinic receptors as a competitive antagonist, it effectively reverses the effect of excessive ACh, thereby improving the symptoms of OP poisoning [10]. Despite its clinical efficacy, ATR mainly provides symptomatic relief and does not directly mitigate the cellular injury, inflammation, or oxidative stress that plays a dominant role in CPF‐induced neurotoxicity and testicular toxicity. This highlights the need for exploring adjunct therapies, which have antioxidant and neuroprotective properties.

*Nigella sativa (N. sativa)* (NS), referred to as black seed or black cumin, has been used in traditional medicine for millennia to treat a wide range of ailments, ranging from digestive and respiratory problems to topical skin disease [11–15]. Thymoquinone, a bioactive compound found abundantly in these seeds, due to its strong antioxidant potential, is thought to be a primary contributor to its pharmacological effects [16–18]. While the exact process by which various components of NS work are still under investigation, studies suggest that it may exert antioxidant, anti‐inflammatory, antimicrobial, analgesic, antidiabetic, anticancer, bronchodilator, spasmolytic, renal protective, hepato‐protective, and gastro‐protective effects [1, 11, 19–22].

The available literature on NS focuses on its protective effects on various toxicities [23–26]. However, studies evaluating the role of NS against CPF‐induced neuro‐ and testicular toxicity remain limited and inconclusive. Hence, in view of the paucity of the studies in this field, we plan to investigate the neuroprotective and antioxidant effects of the ethanolic seed extract of NS against CPF‐induced neurotoxicity and testicular toxicity. It is hypothesized that addressing these gaps will help identify potential therapeutic agents capable of ameliorating oxidative damage caused by OP exposure, thereby providing valuable insights into environmental and reproductive health.

## 2. Materials and Methods

### 2.1. Ethics Approval

The study was conducted in compliance with all applicable regulations concerning animal experiments, and approval was obtained from Institutional Animal Ethics Committee (IAEC/KMC/47/2023) and Institutional Biosafety Committee.

### 2.2. Animals

Male Wistar rats weighing between 180 and 250 g were selected and kept at the central animal house of the institute at Manipal. The animals were kept in cages in rooms maintained at temperature 25 ± 1°C and humidity 50%–55% with a 12‐h light–dark cycle. The animals were fed a conventional rat pellet diet and had unlimited access to water. After a period of acclimatization of 1 week, the respective rats were used for the experiments.

### 2.3. Chemicals

The chemicals used in the study were purchased from Thermo Fisher Scientific, Gennext Scientific & IT Solutions, Shri Durga Laboratory Equipment Supplies, Takara, and Sigma. ATR was acquired from Kasturba Medical College pharmacy, Manipal. NS was bought from the local market in Udupi and validated by a botanist from a teaching institution.

### 2.4. Preparation of Ethanolic Seed Extract of NS

After being dried and milled into a powder, the NS seeds were extracted with 99.9% ethanol using a Soxhlet system to produce an ethanolic extract. The final product was filtered, vacuum‐condensed at 50°C in a rotary evaporator, and then dried at the same temperature in an oven [27].

### 2.5. Gas Chromatography–Mass Spectrometry Analysis (GC‐MS)

A pinch of the extract was dissolved in 2 mL of ethanol, following which the solution was filtered through 0.45‐micron syringe filter, which was then subjected to GCMS analysis. GC in conjunction with a Shimadzu mass spectrometer (MS) (GC/MS—QP2020 NX SHIMADZU, Shimadzu Corp., Tokyo, Japan) was used to analyze the material. For the chromatographic analysis, a GC fitted with a capillary column SH‐I‐5Sil MS Capillary, 30 m × 0.25 mm *x* 0.25 μm, was utilized. With a total flow of 50 mL/min and a column flow of 1.69 mL/min (helium gas), the injector temperature was 2500°C (splitless mode). The oven temperature program was started at 50°C and held there for 1 min. It was then further increased to 140°C at a pace of 20°C per minute, followed by 2800°C at a rate of 10°C per minute, held for 3 min, with a total run of 22.50 min.

### 2.6. Study Design

In the study, animals were divided into six groups of six animals each after acclimatization; 7.5 mg/kg of CPF, a crystalline solid, was mixed with corn oil and given orally [28]; 2.5, 5, and 10 mg/kg of ethanolic extract of NS were dissolved in distilled water and administered orally [29]; and 10 mg/kg of ATR was given intraperitoneally [30]. The six groups were as follows:

 Group 1: Control group (distilled water)
 Group 2: CPF 7.5 mg/kg
 Group 3: CPF 7.5 mg/kg + ATR 10 mg/kg
 Group 4: CPF 7.5 mg/kg + NS 2.5 mg/kg
 Group 5: CPF 7.5 mg/kg + NS 5 mg/kg
 Group 6: CPF 7.5 mg/kg + NS 10 mg/kg

The animals were dosed every day for 28 days, following which they were euthanized using xylazine 10 mg/kg and ketamine 100 mg/kg. Brain, testes, and epididymis were collected after dissection and weighed. For biochemical testing, four animals per group were selected and their brain tissues were frozen at −80°C. Cauda was separated from the testes in two animals per group and sent for further analysis. Testes and brain tissues from two animals per group were preserved in 10 mL of 10% formalin for histopathological examination.

### 2.7. Parameters Measured

1. *Malondialdehyde (MDA)*: MDA levels were determined using the thiobarbituric acid (TBA) reaction. Lipoproteins were precipitated with trichloroacetic acid (pH 2–3) and then heated with TBA. This reaction leads to the formation of MDA‐TBA2, resulting in a pink coloured complex. The amount of MDA was then measured using a spectrophotometer at 532 nm; 1000 µL of 0.67% TBA and 500 μL of 20% TCA were added to 100 µL of the test sample (brain tissue) taken in a test tube. The mixture was incubated for 20 min at 100°C and then cooled under running water. After transferring the contents to an Eppendorf tube, it was centrifuged for 5 min at 12,000 rpm. The supernatant’s absorbance at 532 nm was measured in comparison to a water blank [31].
2. *Glutathione reductase (GSH)*: A redox interaction between GSH and 5,5‐dithiobis (2‐nitrobenzoic acid) (Ellman’s Reagent, DTNB) develops a yellow colour when added to sulphydryl compounds. At 412 nm, the reaction was measured; 200 µL of tissue (brain) supernatant was thoroughly combined with 3 mL of precipitating reagent and 1.8 mL of distilled water. It was then allowed to stand for 5 min before being filtered. Two test tubes bearing the labels blank and test were removed. For the test, 2 mL of the clear filtrate from the aforesaid mixture were added to 8 mL of disodium phosphate buffer, and 1 mL of the DTNB reagent was added as well. The colour developed instantly and stayed steady for 10 min. Using 2 mL of distilled water, 8 mL of phosphate buffer, and 1 mL of DTNB, a reagent ‘blank’ was made. At 412 nm, measurements were made in the spectrometer [32].
3. *Determination of AChE activity*: First, 1 mL of phosphate buffer was used to homogenize 0.1 g samples of brain tissue, and then the samples were centrifuged at 3000 × g for 15 min; 10 μL of the homogenized samples was added to 3 mL of DTNB solution (25 mM DTNB in 75 mM phosphate buffer) to measure the amount of AChE inhibition. After adding 10 µL of 3 mM ACh iodide, the absorbance change at 412 nm was measured using a two‐fold spectrophotometer [29, 33].
4. *Determination of inflammatory markers (ILβ)*: The brain samples were collected in Trizole and kept at −80°C. Trizole was used to extract RNA, and a NanoDrop‐1000 spectrophotometer was used to evaluate the concentration and purity of the material. Each sample’s aliquot of 150 μg RNA was transcribed to cDNA using a SuperScript III kit (Takara Japan). Primers of TNFα and ILβ were designed by NCBI Primer Blast. In the current study, the gene expression of Ilβ was normalized using GAPDH. Using the Syber Green Master, the qPCR reaction was run with 150 ng of cDNA, 0.4 μM of each forward and reverse primer, in a final reaction volume of 10 µL. The initial phase of the PCR thermal cycle was conducted at 95°C for 30 s, followed by 40 cycles of 95°C for 5 s and 60°C for 30 s. The melting curve was examined at 95°C for 15 s. The 2^DDCT^ approach, as outlined by Livak and Schmittgen [44], was used to assess the relative quantities of mRNA for each chosen gene. GAPDH primers were used to normalize all expression levels (Table 1). Every real‐time PCR reaction was carried out in triplets and data were normalized [34].
5. *Sperm density assessment*: Cauda epididymis was removed and put in 2 mL of Earle’s balance salt solution with added 0.1% bovine serum albumin. It was carefully teased with the aid of bent needle, and after observing under a stereomicroscope, a clutch of spermatozoa was released. Sperm density was counted by placing 10 µL of the sperm suspension on Makler’s counting chamber and then counting under light microscope with 10x objective. The total number of sperms present in 100 squares were counted, and the concentration was calculated as millions/mL [35].
6. *Sperm motility assessment*: About 10 µL of the sperm suspension was introduced on a clean microscope slide with a coverslip on it. The percentage of live sperm, viability, and sperm motility were analyzed using a light microscope with an objective lens 40X. These included total motile spermatozoa, which was the number of progressive and nonprogressive motility counted in 10 fields and expressed as percent motile spermatozoa [35].
7. *Spatial memory assessment using Morris water maze (MWM)*: This test uses the animals’ natural need to get out of the water to find the standing platform in order to get them to learn and memorize. In the MWM, rats were tested on their ability to learn spatially by having to travel from start sites around the perimeter of an open swimming arena to a submerged escape platform using distal cues. Repetition of trials was used to evaluate spatial learning, and preference for the platform region in the absence of the platform determined reference memory. Shift and reversal trials improve the ability to identify spatial abnormalities. Four successive acquisition trials were given to each rat for 4 days starting from day 24. During each training session, the latency of rats to locate the hidden platform was recorded. On the 5th day (probe trial day), the hidden platform was taken out and the rats were allowed to explore the pool. The percentage of time spent and the percentage of distance travelled by the rat in target zone were recorded as an indicator of retrieval [36].
8. *Histopathological examination*: After sacrificing the rats, brain and testes were dissected and stored in formalin and were further sent for histopathological sectioning. The brain sections were stained with cresyl violet to determine neurodegeneration, and the testes were stained using hematoxylin and eosin. H&E‐stained tissues were qualitatively examined using a light microscope with a 40 × 10x magnification [37].

**TABLE 1:** Sequence of primers used in real‐time PCR.

Gene name	Primer sequence
GAPDH	F: AGGTCGGAGTCAACGGATTT
	R: ATGAAGGGGTCATTGATGGCA

ILβ	F: AGGCTTCGAGATGAACAACA
	R: GCTCATGGAGAATACCACTTG

## 3. Results

### 3.1. GC‐MS Analysis

**FIGURE 1:**
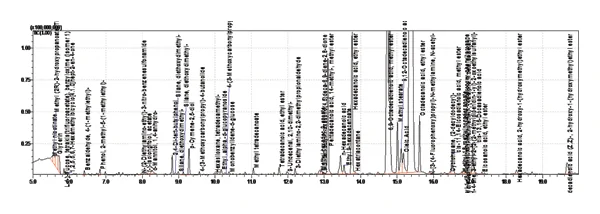
Chromatogram of high‐peak‐intensity compounds in the methanol extract of *N. sativa*.

The GC‐MS analysis of ethanolic seed extract of NS identified several major compounds based on their retention times and peak areas. A total of 128 compounds were recognized by the library. Further, auto‐integration of top 50 peaks (based on height) was performed. Pentadecanoic acid, 14‐methyl‐methyl ester, hexadecanoic acid, ethyl ester, 6,9‐octadecadienoic acid, methyl ester, octadecanoic acid, ethyl ester, methyl stearate, 2,4‐di‐tert‐butylphenol, and oleic acid were some of the compounds identified, among which hexadecanoic acid and ethyl ester recorded the highest peak area (29,705,348) and a retention time of 13.762 min, establishing it as the most abundant constituent of the extract. 6,9‐Octadecadienoic acid followed closely with a peak area of 29,284,590 at a retention time of 14.734 min, indicating its significant presence in the extract. Pentadecanoic acid, 14‐methyl‐methyl ester, showed a peak area of 26,463,825 and a retention time of 13.099, indicating the third most abundant compound in the extract (Figure 1).

### 3.2. Determination of MDA and GSH Levels

There was an increase in MDA levels in groups treated with CPF when compared with the Control and a decrease in GSH levels in groups treated with CPF when compared with the Control and CPF + ATR group. A dose‐dependent decrease and increase in MDA and GSH levels simultaneously was observed in groups treated with different doses of NS along with CPF (Table 2).

**TABLE 2:** MDA and GSH levels in rats of different groups.

Groups	MDA (μmol/L)	GSH (mmol/L)
Control	0.25 ± 0.019	0.058 ± 0.001
CPF	0.775 ± 0.067^a^	0.039 ± 0.001^a^
CPF + ATR	0.403 ± 0.50^b^	0.051 ± 0.002^ab^
CPF + NS(2.5)	0.583 ± 0.061^ac^	0.045 ± 0.002^ab^
CPF + NS(5)	0.397 ± 0.096^bd^	0.050 ± 0.001^ab^
CPF + NS(10)	0.096 ± 0.038^bcde^	0.061 ± 0.002^bcde^

*Note: p* < 0.05, two‐way ANOVA; Tukey multiple comparison test. Mean ± SD was used to report all data.

^a^ *p* < 0.05 vs control.

^b^ *p* < 0.05 vs CPF.

^c^ *p* < 0.05 vs ATR.

^d^ *p* < 0.05 vs CPF + NS(2.5).

^e^ *p* < 0.05 vs CPF + NS(5).

### 3.3. Determination of AChE Activity

AChE activity had a significant decrease in the CPF group compared to Control and CPF + ATR. The activity of CPF + NS(2.5), CPF + NS(5), and CPF + NS(10) was comparable with Control and CPF + ATR, but in comparison with the CPF group, there was an elevation in AChE activity (Table 3).

**TABLE 3:** AChE activity among rats of different groups.

Groups	AChE activity (M/min/mg protein)
Control	0.031 ± 0.0025
CPF	0.016 ± 0.001^a^
CPF + ATR	0.031 ± 0.002^b^
CPF + NS(2.5)	0.027 ± 0.001
CPF + NS(5)	0.026 ± 0.001
CPF + NS(10)	0.030 ± 0.002^b^

*Note: p* < 0.05, two‐way ANOVA; Tukey multiple comparison test. Mean ± SD was used to report all data.

^a^ *p* < 0.05 vs control.

^b^ *p* < 0.05 vs CPF.

### 3.4. Determination of Inflammatory Markers

**FIGURE 2:**
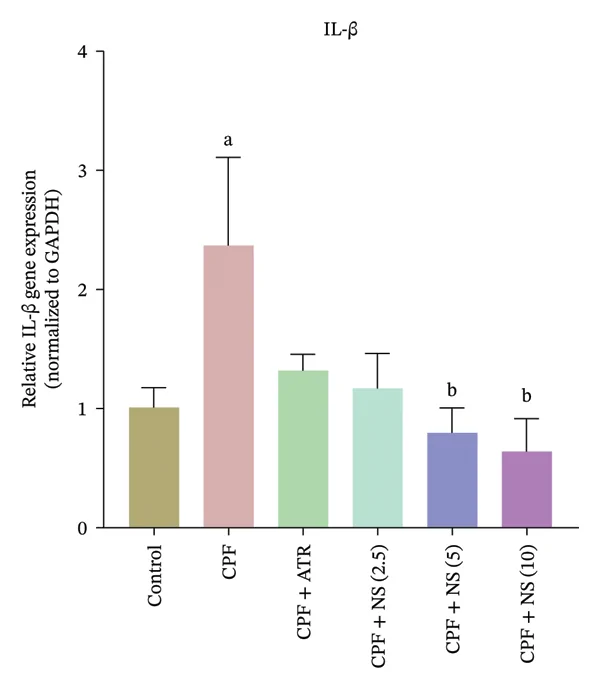
IL‐β levels in rats of different groups. *p* < 0.05, two way ANOVA; Tukey multiple comparison test, ^a^ *p* < 0.05 vs control, ^b^ *p* < 0.05 vs CPF, ^c^ *p* < 0.05 vs ATR, ^d^ *p* < 0.05 vs CPF + NS(2.5), ^e^ *p* < 0.05 vs CPF + NS(5), mean ± SD was used to report all data.

The CPF group showed an increase in IL‐β compared to Control and CPF + ATR groups. The groups CPF + NS(5) and CPF + NS(10) showed a significant decrease in IL‐β levels compared to the CPF group (Figure 2).

### 3.5. Spatial Memory Assessment Using MWM

**FIGURE 3:**
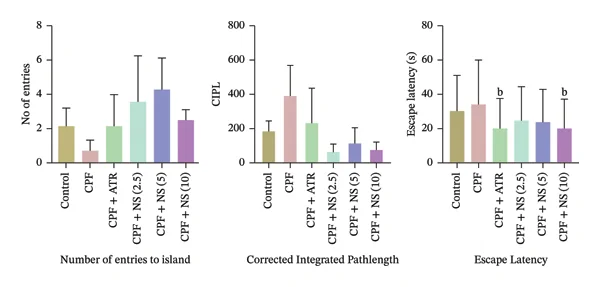
Spatial memory assessment using Morris Water Maze. Spatial memory was evaluated using the parameters: number of entries to the island, corrected integrated path length (CIPL), and escape latency. *p* < 0.05, two‐way ANOVA; Tukey multiple comparison test, ^a^ *p* < 0.05 vs control, ^b^ *p* < 0.05 vs CPF, ^c^ *p* < 0.05 vs ATR, ^d^ *p* < 0.05 vs CPF + NS(2.5), ^e^ *p* < 0.05 vs CPF + NS(5), mean ± SD was used to report all data.

In the CPF group, there are fewer entries to the island compared to all other groups and is much more in the CPF + NS(5) group; however, it is statistically not significant. The corrected integrated path length (CIPL) in the CPF group was much higher than those in other groups, but was not statistically significant. There has been an increase in escape latency of CPF group, and the difference is statistically significant when compared to the CPF + ATR and CPF + NS(10) groups (Figure 3).

### 3.6. Sperm Count/Sperm Motility

Sperm count in CPF group has decreased significantly when compared with the Control, CPF + ATR, CPF + NS(5), and CPF + NS(10) groups. But progressive and nonprogressive motile count in the CPF group has increased when compared to other groups except the CPF + NS(10) group. The nonmotile sperm count was less in the CPF group compared to other groups, except CPF + ATR (Table 4).

**TABLE 4:** Sperm motility and sperm count of various groups.

Groups	Sperm count (M/mL)	Progressive motile (%)	Nonprogressive motile (%)	Nonmotile (%)
Control	61 ± 4.24	1.5 ± 0.71	31 ± 1.41	67.50 ± 2.12
CPF	47 ± 1.41^a^	4 ± 1.41	38.50 ± 3.54	57.5 ± 4.94
CPF + ATR	56.50 ± 0.71^b^	3.5 ± 0.71	42 ± 2.82^a^	54.50 ± 2.12^a^
CPF + NS(2.5)	53.50 ± 1.41	0^b^	25.50 ± 2.12^bc^	74.50 ± 2.12^bc^
CPF + NS(5)	59 ± 2.12^b^	0.5 ± 0.71	30 ± 0.71^c^	69 ± 1.41^bc^
CPF + NS(10)	55.50 ± 4.94^b^	6 ± 1.41^a^	47.50 ± 2.12^a^	61.6 ± 10.19^ab^

*Note: p* < 0.05, one‐way ANOVA; post hoc Tukey test. Mean ± SD was used to report all data.

^a^ *p* < 0.05 vs control.

^b^ *p* < 0.05 vs CPF.

^c^ *p* < 0.05 vs ATR.

### 3.7. Histopathological Evaluation of Brain

Coronal sections of brain were stained with cresyl violet, and qualitative examination of the hippocampal CA3 region’s pyramidal neuronal cell bodies was made under a light microscope. Neurons with a noticeable nucleus, transparent cytoplasm, and a healthy‐looking cell membrane were considered as normal healthy neurons, and neurons with darkly basophilic, flame‐shaped, pyknotic cell bodies were considered as degenerating neurons. Darkly basophilic flame‐shaped cell bodies are suggestive of karyopyknosis in the hippocampal pyramidal neurons.

**FIGURE 4:**
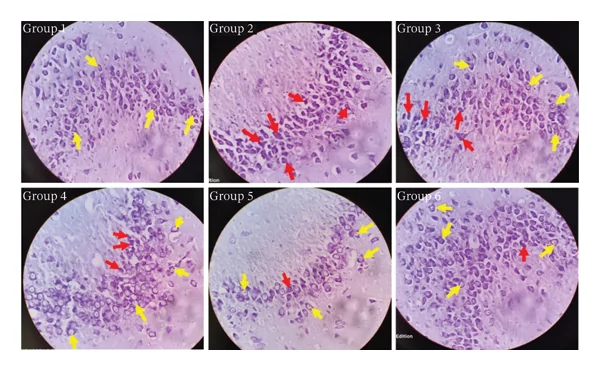
Photomicrographs of the CA3 area of the hippocampus stained with cresyl violet (magnification: 40 × 10 X). Group 1—control, Group 2—CPF, Group 3—CPF + ATR, Group 4—CPF + NS(2.5), Group 5—CPF + NS(5), Group 6—CPF + NS(10).

In the hippocampus CA3 region, a sizable number of degenerating pyramidal neurons, flame‐shaped, pyknotic cell bodies were seen in rats belonging to the CPF group (Group 2) (shown by a red arrow in Figure 4). However, there was an increase in the number of healthy neurons (indicated by yellow arrow) in groups 3, 4, 5, and 6, when compared to Group 2, and is almost on par with the Control group (Group 1). A decrease in number degenerating pyramidal neuronal cell bodies (depicted by red arrow) in hippocampal CA3 region in rats of groups 3, 4, 5, and 6 is noted compared to Group 2 (Figure 4).

### 3.8. Histological Evaluation of Testes

**FIGURE 5:**
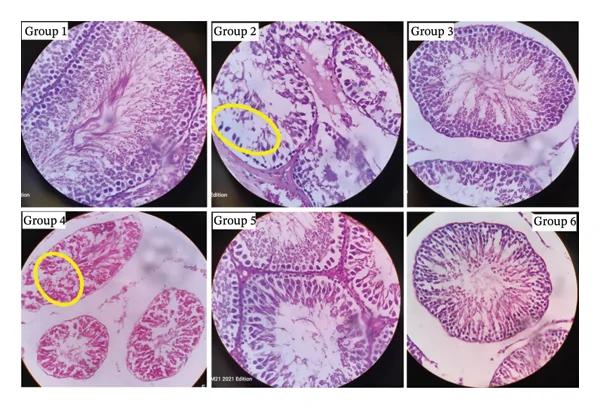
Photomicrographs of hematoxylin and eosin‐stained transverse sections of seminiferous tubules (magnification: 40 × 10 X). Group 1‐control, Group 2‐CPF, Group 3‐CPF + ATR, Group 4‐CPF + NS(2.5), Group 5‐CPF + NS(5), Group 6‐CPF + NS(10).

Qualitative analysis of hematoxylin and eosin‐stained transverse sections of seminiferous tubules of rats of Control group (Group 1) showed typical features of the tubules with strata of spermatogonia at different stages, thick collection of sperms in the lumen and normal‐looking Sertoli cells and basement membrane. The CPF and CPF + NS(2.5) groups show toxicity‐induced damage to the seminiferous tubules, including reduced number of germ cells, and markedly reduced sperms and spermatids along with detachment of germ cells from the basement membrane (yellow encircled region shows the areas with reduced number of germ cells and sperms). However, the structure of seminiferous tubules of Groups 3, 5, and 6 showed recovery and looked normal and close to that of Control group (Group 1) (Figure 5).

## 4. Discussion

This study investigates the effect of NS ethanolic extract on CPF‐induced neurotoxicity and testicular toxicity. CPF was given at 7.5 mg/kg to Wistar albino rats weighing approximately 180–250 g for 28 days, and three different doses of NS extract (2.5, 5, 10 mg/kg) were administered alongside CPF. After 28 days of dosing, the rats were euthanized.

The dose of CPF used in the present study was chosen based on previous experimental studies demonstrating its ability to induce reproductive and oxidative toxic effects in rats without causing significant mortality. CPF was administered orally at a dose of 7.5 mg/kg body weight for 28 consecutive days, as this dose has been reported to produce testicular degeneration, altered spermatogenesis, oxidative stress, and neurobehavioural changes in Wistar rats following subacute exposure [28]. Joshi et al. demonstrated significant testicular toxicity and impairment of spermatogenesis in albino rats treated with CPF at 7.5 mg/kg body weight, supporting the suitability of this dose for evaluating reproductive toxicity. The duration of 28 days was chosen to mimic repeated subacute exposure and to assess the protective efficacy of NS during continued CPF administration. The doses of NS ethanolic extract (2.5, 5, and 10 mg/kg body weight) were selected to evaluate dose‐dependent protective effects against CPF‐induced toxicity [29, 38].

The GC‐MS profile obtained in this study indicated that the ethanolic extract of NS is rich in phenolic compounds and biologically active fatty acid esters, which may potentially contribute to its antioxidant, cytoprotective, and anti‐inflammatory properties against testicular toxicity and neurotoxicity induced by CPF. The most abundant compound in the ethanolic extract was hexadecanoic acid, ethyl ester (ethyl palmitate), which had the largest peak area (29,705,348) with a retention period of 13.762 min (Figure 1). Derivatives of hexadecanoic acid are known to have hypocholesterolemic, antimicrobial, anti‐inflammatory, and antioxidant properties. 6,9‐Octadecadienoic acid, methyl ester, with a peak area of 29,284,590 at RT 14.734 min, was the second major compound identified. This being a member of the unsaturated fatty acid esters class has demonstrated antioxidant, anti‐inflammatory, and membrane‐protective properties. These properties in NS could potentially protect against toxicity caused by CPF [39]. Pentadecanoic acid, 14‐methyl‐methyl ester, recorded a peak area of 26,463,825 at RT 13.099 min, making it the third most abundant constituent in the extract. Branched‐chain fatty acid esters have been reported to exhibit antimicrobial and antioxidant‐related activities, which may contribute to the biological potential of the extract [40]. Another notable compound identified was 2,4‐di‐tert‐butylphenol, a bioactive phenolic compound with well‐documented antioxidant, antimicrobial, antifungal, and anticancer properties. Previous studies have shown that this compound scavenges free radicals and inhibits oxidative damage, suggesting a possible role in the protective effect of NS extract observed in the present study [41, 42].

A study conducted by Abd El‐Moneim Ibrahim et al. on combined or single exposure of cypermethrin and CPF in rats showed oxidative injury through increased MDA and nitric oxide levels. In this study, MDA levels increased and GSH levels dropped dramatically in the CPF group when compared to Control, CPF + ATR, CPF + NS(2.5), CPF + NS(5), and CPF + NS(10) groups (*p* < 0.05) (Table 2). The rise in MDA is attributed to oxidative degeneration in the lipid, especially polyunsaturated fatty acid and free‐radical‐scavenging thiol group [43]. Mahmoud et al. conducted a study on the impact of N‐acetylcysteine on CPF neurotoxicity in rats where decreased glutathione content and increased levels of nitric oxide and MDA were observed. CPF decreases glutathione levels by inducing oxidative stress, inhibiting antioxidant enzymes, disrupting GSH synthesis, and promoting LPO, leading to increased cellular damage [44].

Mesallam et al. carried out studies on propolis and NS oil to counteract the negative effects of CPF on liver and testes of adult albino rats, and the results obtained showed that administration of NS alone in CPF‐treated rats possessed the ability to practically restore MDA levels. A study on the anti‐inflammatory and antioxidant properties of lycopene and thymoquinone on toxicity induced by CPF revealed that administration of NS led to reduction in MDA levels and rise in GSH, GPx, SOD, and CAT; most of the proposed mechanism for antioxidant activity of thymoquinone are closely associated with its direct interaction with GSH or NADP, forming complexes that are capable of scavenging ROS and activating antioxidant genes preferentially [45–47]. Similarly, Öz et al. reported that dietary supplementation with NS oil significantly mitigated diazinon‐induced impairments in growth performance, hematological parameters, and liver function in Nile tilapia, while enhancing antioxidant enzyme activity (SOD, CAT, GPx) and reducing gill and liver tissue damage. These actions have been largely attributed to its bioactive phytoconstituents thymoquinone, further supporting the protective, antioxidant‐mediated role of NS against OP‐induced toxicity [48]. This is consistent with another study done by Öz et al., who demonstrated that NS oil not only confers hemato‐protective and antioxidant protection against boric acid–induced toxicity in Nile tilapia via enhanced CAT, SOD, and GPx activity, but also reduced MDA levels [49]. In this study, the MDA levels decreased and GSH levels increased significantly in CPF + NS(2.5), CPF + NS(5), and CPF + NS(10) groups compared to the CPF group (*p* < 0.05) (Table 2).

According to the study done by Cardona et al., a dose‐dependent decline was observed in AChE activity in the brain without causing cell death on exposure to CPF [50]. The current study exhibited a significant reduction in AChE levels in the CPF group compared to the Control, CPF + ATR, CPF + NS(2.5), CPF + NS(5), and CPF + NS(10) groups, indicating CPF‐induced neurotoxicity(*p* < 0.05) (Table 3). By inhibiting a membrane‐bound enzyme AChE, which is necessary for AChE metabolism at neuromuscular junctions and cholinergic synapse, CPF acts as a neurotoxin [51]. By phosphorylating AChE’s serine OH group, CPF permanently inhibits the enzyme, which results in cholinergic hyperstimulation and ACh accumulation in the brain, which can lead to a wide range of clinical conditions [50, 52]. Abdel‐Rahman M conducted a study in rats to determine the activity of NS on ciprofloxacin and pentylenetetrazol‐treated rats where NS was successful in restoring the monoamine levels and AChE activity in the cerebellum, hippocampus, and cerebral cortex [53]. When NS and DDVP were administered simultaneously in an investigation carried out by Aminu et al. on the protective effect of NS oil on memory impaired rats due to oxidative dysfunction, it became clear that NS could protect the hippocampal cholinergic integrity by reducing the amount of AChE deactivation caused by DDVP in the exposed animals. These reports have been linked to memory function tasks and cholinergic interactions, which may explain NS’s efficacy against neuropsychiatry, neurocognitive, and neurodegenerative impairments [54]. This research found that NS was successful in restoring the AChE activity in the CPF + NS(2.5), CPF + NS(5), and CPF + NS(10) groups. The CPF + NS(10) group also demonstrated a notable rise in AChE activity when compared with the CPF group (*p* < 0.05) (Table 3).

A study conducted by Owumi SE and DimUJ on the effect of manganese on CPF exposure showed increased pro‐inflammatory cytokines TNFα and IL‐β levels on exposure to CPF. This may be related to NF‐Κb activation mediated by ROS [55]. Umar et al. conducted a study on thymoquinone action on collagen‐induced arthritis, which showed that thymoquinone oral administration resulted in decreased levels of inflammatory mediators, including PGE2, TNFα, ILβ, IL‐6, and IFN‐C, attributed to mechanisms including inhibition of proinflammatory cytokines, reduced free radical production, and LPO suppression, thereby improving the antioxidant defence system [56]. Mihaylova et al. investigated the effect of NS oil on rat memory function and serum levels of inflammatory cytokines with lipopolysaccharide‐induced neuroinflammation, in which there was a significant decrease in IL‐1β levels among others displaying cognitive improvement and anti‐inflammatory effect [57]. The current study showed that there was a significant dose‐dependent decrease in ILβ levels in the CPF + NS(2.5), CPF + NS(5), and CPF + NS(10) groups in contrast to the CPF group (*p* < 0.05) (Figure 2). CPF‐mediated mitochondrial oxidative stress might trigger inflammation and innate immune response, raising the possible role of ROS scavengers in reversing pesticide‐related damage [58].

A study conducted by Lopez‐Granero et al. on the long‐term spatial memory impairment and thigmotaxic behaviour showed increased thigmotaxis, or a tendency for swimming close to pool walls, in CPF dietary group, suggesting elevated anxiety or fear. Additionally, they spent less time in the vicinity of the previously situated platform, indicating a decrease in spatial bias and a potential impairment in long‐term spatial memory. Disruptions in glutamatergic and/or GABAergic systems, which are critical for the best possible behaviour, motor functions, and cognition, may be the cause of these emotional and cognitive changes [59]. Similarly, in the present study, rats exposed to CPF showed fewer entries to the island and an increased CIPL, indicating impaired spatial memory and difficulty utilizing spatial cues. Elevated escape latency and enhanced thigmotaxis further supported the cognitive deficits associated with CPF exposure. Imam et al. conducted a study on cannabis‐induced moto‐cognitive dysfunction in Wistar rats to test ameliorative efficacy of NS, which showed improved cognitive function by reducing latency in locating a hidden platform [60]. In the current study, there has been an increase in the number of entries to the island in groups treated with different doses of NS along with CPF, and there was a decrease in CIPL and escape latency in these groups (Figure 3).

A study conducted by Badazadeh and Najafi on sperm characteristics and testicular changes on CPF exposure resulted in oxidative stress‐mediated sperm damage and decrease in sperm production [61]. CPF interferes with sperm maturation and motility and causes alteration such as Leydig cell drop and negative tubular differentiation. Sperm quality is further compromised by increased DNA damage, which affects motility and count. Leisegang et al. used an obese animal model to determine the effect of NS oil on mitochondrial membrane potential, serum testosterone, and semen parameters to reveal improved sperm concentration and testosterone levels along with a significant increase in the percentage of mitochondrial membrane potential intact sperm [62]. In our study, the sperm count decreases significantly in CPF groups when compared with the Control, CPF + ATR, CPF + NS(5), and CPF + NS(10) groups (*p* < 0.05) (Table 4). However, there was an increase in progressive motile and nonprogressive motile count and a reduction in nonmotile sperm count in the CPF group than in the Control group, which was not statistically significant. This dissociation between sperm count and motility suggests that spermatogenesis is more affected by CPF toxicity than motility mechanisms. This may also reflect the kinetics of the exposure window relative to the spermatogenic cycle: since spermatogenesis in rats spans approximately 50 days and epididymal transit (during which motility is acquired) requires a further 10 days. Since epididymal sperm maturation is a multiday, region‐dependent process (caput, corpus, cauda) compared to testicular spermatogenesis, sperm which was sampled at the end of a subacute CPF exposure period is likely to represent a mixed population consisting of some that had already completed epididymal maturation prior to peak testicular injury, and a noticeably reduced cohort of newly forming germ cells that were highly vulnerable to CPF‐induced blood–testis barrier disruption and oxidative injury. This apparent count‐motility dissociation would preferentially deplete sperm number while relatively sparing the motility phenotype of the surviving, already‐matured population which is consistent with selective survival rather than a true improvement in motility mechanisms [63, 64].

Rat testicular histopathological alterations were significantly improved by NS extract. A study done by Mosbah et al. on the defensive characteristics of NS for reproductive toxicity due to CPF showed that NS mitigates degenerative changes in seminiferous tubules, restores normal architecture, and enhances spermatogenesis. NSO’s ability to scavenge free radicals and boost antioxidant enzymes in rats is the mechanism by which it protects against the harmful effects of CPF. Histological examinations show increased thickness of seminiferous epithelium, improved density of germ cells, and reduced vacuolation and degeneration in testicular tissue, indicating a protective and restorative effect on testicular health [65]. In the present study, transverse section of rat seminiferous tubules revealed characteristic features of the tubules, including basement membrane and Sertoli cells that appear normal, thick collection of sperm in the lumen, and layers of spermatogonia at various stages. The seminiferous tubules of CPF and CPF + NS(2.5) groups demonstrated toxicity‐induced damage, including a substantial decrease in germ cells and a notable decrease in sperm and spermatid counts (Figure 5).

Earlier studies done on the subchronic neurotoxicity of CPF by Wang et al. and the potential effect of quercetin on CPF‐induced neurotoxicity by Kaur noted that the hippocampal regions of rats given CPF treatment after eosin staining showed necrotic cell characterized by neuronal shrinkage, granular layer disarray, and an apparent loss of pyramidal cells. These modifications show how CPF negatively affects brain cell structure and function, through mechanisms such as oxidative stress and inhibition of mitochondrial enzyme activity, which causes neurotoxicity [66, 67]. Our study showed histopathological findings in CA3 hippocampal region of rats in the CPF group, which had many flame‐shaped, pyknotic, degenerating pyramidal neuronal cell bodies, which is parallel to the findings of previous studies (Figure 4). In a study done by Madkour et al. regarding NS oil’s protective abilities against neurotoxicity induced by emamectin, the NS group demonstrated normal structure of neurons in the cerebellum and hippocampus and promoted normal cellular density of granular cells of hippocampus and cerebellum [68]. Another study conducted by Oz et al. to investigate the effects of NS oil on cypermethrin‐intoxicated Nile tilapia showed improvement of negative effects on growth, haematology, and biochemical values along with amelioration of insecticide‐induced toxic effects on liver and brain, as confirmed by histopathology [69]. In the current study, the hippocampus CA3 region of rats in the groups CPF + ATR, CPF + NS(2.5), CPF + NS(5), and CPF + NS(10), showed rise in healthy neurons and a reduction in pyramidal neuronal cell bodies that were degenerating (Figure 4).

## 5. Conclusion

The results of the present study indicate that the ethanolic seed extract of NS has significant neuroprotective activity against CPF‐induced neurotoxicity, mainly at the dose of 10 mg/kg, as portrayed by the improvement in AChE activity and cognitive performance combined with its anti‐inflammatory effect through a decrease in IL‐1β levels and antioxidant effect through an increase in GSH and reduction in MDA levels. However, the NS extract did not show any significant protective effect against CPF‐induced testicular toxicity. These results indicate the potential therapeutic role of NS in mitigating OP‐induced neurological damage, justifying further studies to elucidate its mechanisms and extended benefits.

## Author Contributions

Concept and design: Dhanya R. Rai, Veena Nayak, Reena Sherin Parveen, Sandhya Kumari, and Vinutha R. Bhat.

Data acquisition: Dhanya R. Rai, Reena Sherin Parveen, Veena Nayak, Sandhya Kumari, Vinutha R. Bhat, and Harshini.

Data analysis/interpretation: Dhanya R. Rai, Reena Sherin Parveen, Veena Nayak, Sandhya Kumari, Vinutha R. Bhat, Harshini, and Mohandas Rao K. G.

Drafting manuscript: Dhanya R. Rai, Reena Sherin Parveen, Veena Nayak, Sandhya Kumari, Vinutha R. Bhat, Harshini, and Mohandas Rao K. G.

Critical revision of manuscript: Dhanya R. Rai, Reena Sherin Parveen, and Veena Nayak.

Statistical analysis: Dhanya R. Rai, Reena Sherin Parveen, Veena Nayak, and Sandhya Kumari.

Funding: Reena Sherin Parveen, and Veena Nayak.

Admin, technical or material support: Reena Sherin Parveen, Veena Nayak, Sandhya Kumari, Vinutha R. Bhat, and Mohandas Rao K. G.

Supervision: Reena Sherin Parveen, Veena Nayak, and Sandhya Kumari.

## Funding

The authors received no specific funding from any funding agency in the public, commercial, or not‐for‐profit sectors.

## Disclosure

All authors approved the final manuscript.

## Conflicts of Interest

The authors declare no conflicts of interest.

## Data Availability

The data that support the findings of this study are available upon request from the corresponding author. The data are not publicly available due to privacy or ethical restrictions.
